# Maternal temperament and character: associations to child behavior at the age of 3 years

**DOI:** 10.1186/s13034-021-00375-5

**Published:** 2021-05-06

**Authors:** Sara Agnafors, Marie Bladh, Lisa Ekselius, Carl Göran Svedin, Gunilla Sydsjö

**Affiliations:** 1 Division of Children’s and Women’s Health, Department of Biomedical and Clinical Sciences, Linköping University, SE-581 85 Linköping, Sweden; 2 Department of Neuroscience, Psychiatry, Uppsala University, SE-751 85 Uppsala, Sweden; 3 Department of Social Science, Ersta Sköndal Bräcke, University College, Stockholm, Sweden

**Keywords:** Children, Mothers, Temperament, Behavior problems, SESBiC

## Abstract

**Background:**

The influence of maternal temperament on child behavior, and whether maternal temperament impact boys and girls differently is not thoroughly studied. The aim was to investigate the impact of maternal temperament and character on child externalizing and internalizing problems at age 3.

**Methods:**

A birth-cohort of 1723 mothers and their children were followed from birth to age 3. At the child’s age of 3 months, the mothers filled out standardized instruments on their temperament and character using the Temperament and Character Inventory (TCI) and depressive symptoms using the Edinburgh Postnatal Depression Scale (EPDS). At the child’s age of 3 years, the mothers reported on child behavior using the Child Behaviour Checklist (CBCL).

**Results:**

Maternal temperamental trait novelty seeking was positively associated with externalizing problems in the total population and in girls. Harm avoidance was positively associated with externalizing problems in the total population and in boys, and with internalizing problems in the total population and boys and girls respectively. Maternal character traits of self-directedness and cooperativeness were negatively associated with both externalizing and internalizing problems in the total population and in boys and girls respectively.

**Conclusions:**

Maternal character traits were more influential on child behavior than were temperamental traits, and thus the opportunities for intervention targeted at parental support are good. Maternal mental health and socioeconomic aspects also increased the risk for child behavior problems, indicating the need for recognition and support in clinical settings.

## Background

Child development is evidently under the influence of parental behavior and family environment. Parental mental state and disorders impact child behavior and the development of child psychopathology [1, 2]. Moreover, parenting styles vary also in healthy individuals, contributing to nourishing as well as detrimental growth environments [3]. Given the impact of parental influence, intrinsic factors such as parental personality and temperament are of interest to understand dysfunctional development in children.

Cloninger’s psychobiological model of personality builds on a dynamic interaction between two domains of personality; temperament and character [4]. The model suggests a multidimensional nature of personality traits and personality dysfunction, accounting for normal as well as abnormal variation in personality. According to Cloninger, temperamental traits determine automatic responses to emotional stimuli, and can be measured as four separate dimensions; novelty seeking, harm avoidance, reward dependence and persistence. While temperamental factors can be identified during early childhood, the character domain of Cloninger’s model of personality, mature during adulthood [4]. The temperamental and character traits of Cloninger’s psychobiological model have been associated with different types of psychopathology in adulthood. High novelty seeking has been linked to ADHD and addiction [5, 6], while high harm avoidance and low self directedness have been associated with many types of mental disorders [7].

Few studies examined the association between parental temperament and child psychopathology in young children. Yurumez et al. (2018) found positive correlations between parental harm avoidance and child attention deficit hyperactivity disorder (ADHD), while negative correlations were found between self-directedness and ADHD [8]. Lee et al. (2015) found the temperamental trait of harm avoidance to be positively, and the character traits of self-directedness and self-transcendence to be negatively associated with behavior problems in pre-school children [9]. Studies using other measures of personality found high parental neuroticism and low conscientiousness to be associated with child antisocial behaviors [10]. Parental personality has also been found to impact parenting style [11], thus parenting style could mediate the association between parental personality and child psychopathology. Studies on older children have shown an association between adolescent suicide attempts and high harm avoidance in mothers and low self-directedness in fathers [12]. In a study on adult patients with borderline personality disorder, mothers of patients were found to display lower levels of self-directedness, while fathers displayed higher levels of novelty seeking, harm avoidance and self-transcendence and lower levels of self-directedness compared to control subjects [13]. Moreover, interactions between parental and child traits might influence effects on child behavior [14, 15]. Rettew et al. (2006), found the combination of high maternal and child novelty seeking to be associated with child attention problems, and similarly high harm avoidance in fathers and children were associated with child internalizing problems [15]. In general, interaction effects were higher than effects of single traits [15]. A recent study examining similar features such as the association between maternal personality and child behavior, found maternal borderline trait to significantly predict internalizing, externalizing and dysregulation problems in pre-school children [16].

### Aim

The aim of the present study was to investigate the impact of maternal temperament and character on child externalizing and internalizing problems in a birth cohort of 1700 children and their mothers (the South East Sweden Birth Cohort (SESBiC) study). This prospective cohort study adds to the field by the use of a large population-based sample, whereas previous studies included smaller, clinical populations [8, 9, 15].We choose to investigate the impact of separate traits in mothers in order to evaluate not only pathologic states, but also the wider range of temperamental traits and related behaviors. Moreover, we control for maternal symptoms of postpartum depression which are known to increase the risk for child behavioral and emotional problems. To our knowledge, the impact of maternal temperament on child behavior in boys and girls respectively has not previously been studied. Given the gender differences in psychopathologic pattern during childhood and adolescence, different susceptibility to plausibly associated factors are of interest to further understand and prevent development of mental health problems in boys and girls respectively.

## Methods

### Subjects

The SESBiC study is based on 1723 mother-child pairs of children born in five adjacent municipalities in southern Sweden during 20 consecutive months in 1995–1996. All mothers of children reported from the delivery wards to Child Welfare Centers (CWC) in the geographically defined catchment area between May 1st 1995 and December 31st 1996 were invited to take part in the study, and 88 % (n = 1723) accepted. At childbirth, the mean age of mothers was 28.2 ± 4.6. The majority (96 %, n = 1574) of mothers were cohabitating, 3.5 % (n = 57) were single parents, and 0.5 % (n = 8) reported other family arrangements. Mothers born in Sweden constituted the majority (n = 1 482, 88.6 %), while 6.2 % (n = 103) were born in Europe, and 5.3 % (n = 88) outside Europe. Of the children, 52.8 % were boys and there were 27 twin pairs. At the 3-year follow-up, one child was deceased. Mothers of 1432 children (83 %) agreed to participate.

### Procedure

The baseline study was carried out at CWCs in connection with the routine 3-month check-up. Information about the study was given by the CWC staff. The mothers were interviewed by a child psychologist and standardized instruments were administered. The 3-year follow-up took place in connection with the routine examination of 3-year-olds at the CWC. Mothers were asked to fill in psychological instruments and questionnaires, and medical information was retrieved from the child’s medical records.

### Instruments

#### Baseline mother assessment

Temperament and Character Inventory (TCI) is an inventory of personality traits developed by Cloninger et al. (1994) [17]. The original version holds 240 items, and the shortened version used in the present study 125 items (TCI-125). TCI evaluates seven components of personality; novelty seeking (NS), harm avoidance (HA), reward dependence (RD) and persistence (PS), self-directedness (SD), cooperativeness (CO) and self-transcendence (ST), the first four constituting temperamental traits and the last three, character traits. Individuals with high novelty seeking are characterized by impulsivity, quick loss of temper and exploratory action. High harm avoidance implicates worrying, shyness, fearfulness and easily fatigue. Individuals with high reward dependence are characterized by warmth, sensitivity, and dependence on social approval. Lastly, high persistence means perseverance despite frustration or fatigue [4]. Character includes three dimensions; self-directedness, cooperativeness and self-transcendence. Self-directedness reflects the ability to regulate and adapt to a given situation, cooperativeness represents the capability of interacting and relating to other people, while self-transcendence reflects individuals’ ability to view themselves as an integral part of the universe [4]. The rank-order stability of the TCI traits has been shown to be fairly high over time in adults [18]. The TCI has been translated into several languages and is widely used, also in Swedish samples [19]. The TCI was answered by the mothers at baseline.

The Edinburgh Postnatal Depression Scale (EPDS) [20] is a well-established self-report form holding 10 items, each rating 0–3 with a total score of 30. The questionnaire is mostly used to screen for postnatal depression in community samples and asks for symptoms experienced during the preceding week. The Swedish version of the EPDS has been validated in community samples [21, 22]. The EPDS was answered by the mothers at baseline.

#### 3-Year follow-up child assessment

The Child Behavior Check List/2–3 (CBCL) [23] is designed to assess child behavior during the last two months and comprises the two main domains of internalizing and externalizing behavioral problems. The form holds 100 items and each item is scored 0–2 from “not a problem” to “often a problem”. The CBCL is widely used and has proven an effective screening tool for child psychiatric disorders [24]. The Swedish version of the CBCL 2/3 was answered by the mothers at the 3-year follow-up.

The Coddington Life Event Scale (CLES) [25] is a 33-item form used to screen for exposure to different life events, (i.e. parental divorce, domestic violence and serious illness or injury within the family). A modified version of the original scale was used, consisting of virtually the same events as the CLES but only evaluating the occurrence of an event and not when it occurred. At the 3-year follow-up, the mothers were instructed to report life events within the family since the birth of the participating child. The CLES is widely used, and similar versions have been used previously in Swedish population-based studies [26].

### Data analysis

To analyze the association between maternal temperamental traits and child behavioral problems, multivariate linear regression was performed with CBCL internalizing and externalizing scales as dependent variables and TCI subscales novelty seeking, harm avoidance, reward dependence, persistence, self-directedness, cooperativeness and self-transcendence, EPDS, life events and sociodemographic covariates (parental divorce, maternal unemployment and parental immigration status) as independent variables. Separate analyses were run for each TCI subscale. Analyses were also run separately for boys and girls to examine whether there were gender differences in the association between child behavior and maternal temperamental and character traits. Drop out analyses were performed using Pearson’s chi-square on baseline information comparing participants to non-participants at the 3-year follow-up. Results are presented with corresponding coefficients (B) and 95 % Confidence Intervals (CI). Statistical significance was adjusted from p-value < 0.05 to a p-value < 0.007 due to multiple testing according to the Bonferroni method (0.05/7 = 0.007, as there are seven tests for each of the outcomes). All statistical analyses were performed using IBM SPSS version 26 (IBM Corporation, Armonk, NY). For frequencies see Table 1.

**Table 1:** Frequency characteristics of the study population

Variable	N (%) N = 1432	Median/range or M/SD
*Control variables*		
EPDS	1422	4/0–22
Life events	1426	4.42/2.62
Family status 3 years	1405	
Divorced	141 (8.2 %)	
Maternal employment status 3 years	1418	
Unemployed	146 (8.5 %)	
Parental immigration status	1428	
One or both parents born abroad	163	
*Temperament dimensions*		
Novelty seeking (NS)	1416	10/0–19
Harm aviodance (HA)	1416	7/0–20
Reward dependence (RD)	1416	10/1–15
Persistence (PS)	1416	2/0–5
*Character dimensions*		
Self-directedness (SD)	1416	21/3–25
Cooperativeness (CO)	1416	22/8–25
Self-transcendence (ST)	1416	4/0–15
*Dependent variables*		
CBCL internalizing	1428	3/0–23
CBCL externalizing	1428	7/0–34

*EPDS*  Edinburgh Postnatal Depression Scale, *CBCL* Child Behaviour Checklist, *CO*  cooperativeness, *HA*  harm avoidance, *NS* novelty seeking, *PS*  persistence, *RD*  reward dependence, *SD* self directedness, *ST*  self transcendence

## Results

### Externalizing problems

#### Total sample

Maternal temperamental traits of novelty seeking and harm avoidance predicted child externalizing problems at age 3, adjusting for sociodemographic variables and symptoms of postpartum depression (Table 2). No association was found between reward dependence and persistence and externalizing problems respectively. The character traits self directedness and cooperativeness in mothers, were negatively associated with externalizing problems at age 3 adjusting for the above-mentioned variables. No significance was reached for self-transcendence (Table 3).

**Table 2:** Maternal temperament traits predicting child externalizing problems in the total population and boys and girls respectively. Linear regression

	Total population		Boys		Girls	
	Adjusted B (95.0 % CI)	p-value	Adjusted B (95.0 % CI)	p-value	Adjusted B (95.0 % CI)	p-value
*Maternal temperament trait*						
Novelty seeking (NS)	0.18 (0.08–0.29)	0.001	0.10 (− 0.06–0.25)	0.214	0.27 (0.12–0.42)	< 0.001
EPDS	2.12 (1.02–3.23)	< 0.001	1.37 (− 0.22–2.96)	0.091	3.02 (1.50–4.54)	< 0.001
Life events	4.35 (2.97–5.74)	< 0.001	6.23 (4.39–8.06)	< 0.001	1.17 (− 0.95–3.29)	0.278
Divorce	0.10 (− 1.09–1.28)	0.875	− 0.03 (− 1.71–1.65)	0.971	0.10 (− 1.55–1.75)	0.904
Unemployment	2.77 (1.58–3.95)	< 0.001	2.04 (0.45–3.63)	0.012	3.85 (2.07–5.63)	< 0.001
Parental immigration	0.22 (− 1.05–1.49)	0.734	0.04 (− 1.59–1.67)	0.960	0.08 (− 2.00–2.16)	0.938
Harm avoidance (HA)	0.19 (0.11–0.27)	< 0.001	0.28 (0.16–0.39)	< 0.001	0.11 (0.00–0.22)	0.050
EPDS	1.52 (0.40–2.65)	0.008	0.61 (− 0.98–2.21)	0.453	2.61 (1.04–4.18)	0.001
Life events	4.46 (3.08–5.84)	< 0.001	6.28 (4.47–8.08)	< 0.001	1.33 (− 0.81–3.46)	0.223
Divorce	0.03 (− 1.15–1.21)	0.961	0.05 (− 1.60–1.70)	0.956	0.03 (− 1.63–1.70)	0.968
Unemployment	2.80 (1.62–3.98)	< 0.001	1.95 (0.39–3.52)	0.015	3.93 (2.14–5.72)	< 0.001
Parental immigration	0.20 (− 1.06–1.47)	0.753	0.10 (− 1.50–1.71)	0.902	0.14 (− 1.96–2.24)	0.895
Reward dependence (RD)	− 0.04 (− 0.18–0.09)	0.539	− 0.08 (− 0.28– 0.13)	0.457	− 0.02 (− 0.20– 0.16)	0.826
EPDS	2.11 (1.00–3.23)	< 0.001	1.41 (− 0.19–3.00)	0.084	2.97 (1.43–4.51)	< 0.001
Life events	4.44 (3.05–5.83)	< 0.001	6.27 (4.43–8.11)	< 0.001	1.31 (− 0.83–3.45)	0.228
Divorce	− 0.02 (− 1.17–1.21)	0.972	− 0.08 (− 1.76–1.59)	0.922	0.08 (− 1.60–1.75)	0.929
Unemployment	2.80 (1.61–3.99)	< 0.001	2.08 (0.49–3.68)	0.010	3.88 (2.09–5.68)	< 0.001
Parental immigration	0.19 (− 1.08–1.47)	0.765	− 0.01 (− 1.64–1.62)	0.987	0.22 (− 1.88–2.33)	0.836
Persistence (PS)	0.01 (− 0.23–0.25)	0.919	− 0.17 (− 0.51–0.17)	0.323	0.23 (− 0.11–0.56)	0.185
EPDS	2.09 (0.97–3.21)	< 0.001	1.44 (− 0.16–3.04)	0.077	2.89 (1.35–4.43)	< 0.001
Life events	4.43 (3.03–5.82)	< 0.001	6.33 (4.49–8.18)	< 0.001	1.25 (− 0.89–3.39)	0.252
Divorce	− 0.01 (− 1.20–1.18)	0.989	− 0.04 (− 1.72–1.64)	0.964	0.07 (− 1.60–1.74)	0.933
Unemployment	2.79 (1.60–3.98)	< 0.001	2.09 (0.50–3.68)	0.010	3.87 (2.08–5.67)	< 0.001
Parental immigration	0.20 (− 1.08–1.47)	0.762	0.04 (− 1.60–1.67)	0.963	0.28 (− 1.83–38)	0.797

*CI*  confidence interval, *EPDS*  Edinburgh Postnatal Depression Scale. Multivariate linear regression. Dependent variables: CBCL externalizing scores. Independent variables: Maternal temperamental traits, EPDS, life events, divorce, unemployment, parental immigration status

**Table 3:** Maternal character traits predicting child externalizing problems in the total population and boys and girls respectively. Linear regression

	Total population		Boys		Girls	
	Adjusted B (95.0 % CI)	p-value	Adjusted B (95.0 % CI)	p-value	Adjusted B (95.0 % CI)	p-value
*Maternal character trait*						
Self-directedness (SD)	− 0.35 (− 0.45–− 0.26)	< 0.001	− 0.42 (− 0.56– − 0.28)	< 0.001	− 0.29 (− 0.43– − 0.16)	< 0.001
EPDS	0.88 (− 0.26–2.02)	0.132	− 0.30 (− 1.95–1.35)	0.721	2.11 (0.54–3.67)	0.008
Life events	4.31 (2.94–5.67)	< 0.001	6.16 (4.37–7.95)	< 0.001	1.13 (− 0.97–3.24)	0.291
Divorce	0.45 (− 0.72–1.62)	0.452	0.46 (− 1.18–2.10)	0.583	0.40 (− 1.26–2.05)	0.637
Unemployment	2.77 (1.60–3.93)	< 0.001	2.03 (0.48–3.58)	0.011	3.84 (2.07–5.61)	< 0.001
Parental immigration	− 0.09 (− 1.34–1.17)	0.893	− 0.22 (− 1.81–1.38)	0.791	− 0.18 (− 2.26–1.90)	0.864
Cooperativeness (CO)	− 0.25 (− 0.37– − 0.13)	< 0.001	− 0.24 (− 0.41– − 0.07)	0.006	− 0.28 (− 0.44–− 0.11)	0.001
EPDS	1.90 (0.79–3.01)	0.001	1.19 (− 0.40–2.77)	0.143	2.72 (1.18–4.25)	0.001
Life events	4.49 (3.10–5.87)	< 0.001	6.29 (4.46–8.12)	< 0.001	1.38 (− 0.74–3.50)	0.202
Divorce	0.28 (− 0.91–1.47)	0.640	0.15 (− 1.53–1.82)	0.864	0.42 (− 1.26–2.09)	0.625
Unemployment	2.88 (1.70–4.07)	< 0.001	2.09 (0.51–3.67)	0.010	4.05 (2.27–5.84)	< 0.001
Parental immigration	0.19 (− 1.08–1.45)	0.775	− 0.01 (–1.64–1.61)	0.988	0.16 (− 1.93–2.24)	0.883
Self-transcendence (ST)	0.01 (− 0.10–0.12)	0.840	0.08 (− 0.07–0.24)	0.291	− 0.05 (− 0.21–0.10)	0.491
EPDS	2.09 (0.97–3.20)	< 0.001	1.27 (− 0.33–2.87)	0.119	3.00 (1.46–4.54)	< 0.001
Life events	4.42 (3.02–5.82)	< 0.001	6.17 (4.33–8.02)	< 0.001	1.40 (− 0.75–3.56)	0.202
Divorce	− 0.00 (− 1.19–1.19)	0.996	− 0.09 (− 1.76–1.59)	0.918	0.06 (− 1.61–1.73)	0.943
Unemployment	2.79 (1.60–3.98)	< 0.001	1.99 (0.40–3.59)	0.014	3.89 (2.10–5.69)	< 0.001
Parental immigration	0.18 (− 1.10–1.47)	0.778	− 0.14 (− 1.78–1.51)	0.872	0.26 (− 1.84–2.37)	0.805

*CI* confidence interval, *EPDS*  Edinburgh Postnatal Depression Scale. Multivariate linear regression. Dependent variables: CBCL externalizing scores. Independent variables: Maternal character traits, EPDS, life events, divorce, unemployment, parental immigration status

#### Gender specific analyses

Novelty Seeking in mothers was associated with externalizing problems in girls but not in boys (Table 2). Quite contrary, maternal Harm Avoidance was associated with externalizing problems in boys, but not in girls. No associations were found for Reward Dependence and Persistence. Maternal character traits of Self Directedness and Cooperativeness were negatively associated with externalizing problems in both boys and girls (Table 3).

### Internalizing problems

#### Total sample

In multivariate analysis, maternal harm avoidance increased the risk for child internalizing problems at age 3 (Table 4). Novelty seeking, reward dependence and persistence had no effects on internalizing problems. Regarding maternal character traits, self-directedness and cooperativeness showed a negative association with internalizing problems in children at age 3, adjusting for symptoms of post-partum depression and sociodemographic factors (Table 5). Effects were lower for internalizing problems compared to externalizing problems.

**Table 4:** Maternal temperament traits predicting child internalizing problems in the total population and boys and girls respectively. Linear regression

	Total population		Boys		Girls	
	Adjusted B (95.0 % CI)	p-value	Adjusted B (95.0 % CI)	p-value	Adjusted B (95.0 % CI)	p-value
*Maternal temperament trait*						
Novelty seeking (NS)	0.04 (− 0.02–0.11)	0.157	0.05 (− 0.03–0.14)	0.228	0.04 (− 0.05–0.12)	0.402
EPDS	1.27 (0.63–1.91)	< 0.001	0.38 (− 0.52–1.28)	0.410	2.28 (1.38–3.18)	< 0.001
Life events	1.77 (0.96–2.57)	< 0.001	2.94 (1.90–3.98)	< 0.001	− 0.06 (− 1.31–1.20)	0.927
Divorce	− 0.49 (− 1.17–0.20)	0.161	− 0.25 (− 1.20–0.70)	0.607	− 0.81 (− 1.79–0.17)	0.104
Unemployment	1.27 (0.59–1.96)	< 0.001	0.97 (0.07–1.87)	0.036	1.78 (0.73–2.84)	0.001
Parental immigration	0.43 (− 0.30–1.16)	0.251	0.43 (− 0.50–1.36)	0.362	0.55 (− 0.69–1.78)	0.386
Harm avoidance (HA)	0.11 (0.06–0.16)	< 0.001	0.12 (0.05–0.18)	< 0.001	0.10 (0.04–0.17)	0.003
EPDS	0.94 (0.29–1.59)	0.005	0.05 (− 0.86–0.96)	0.913	1.95 (1.03–2.87)	< 0.001
Life events	1.80 (1.01–2.59)	< 0.001	2.97 (1.94–4.00)	< 0.001	− 0.03 (− 1.27–1.22)	0.968
Divorce	− 0.49 (− 1.17–0.18)	0.154	− 0.23 (− 1.17–0.72)	0.637	− 0.84 (− 1.82–0.13)	0.089
Unemployment	1.28 (0.60–1.96)	< 0.001	0.93 (0.04–1.83)	0.041	1.83 (0.78–2.88)	0.001
Parental immigration	0.43 (− 0.30–1.16)	0.250	0.45 (− 0.47–1.37)	0.337	0.49 (− 0.74–1.71)	0.436
Reward dependence (RD)	− 0.08 (− 0.16– − 0.01)	0.036	− 0.08 (− 0.20– 0.04)	0.171	− 0.08 (− 0.19– 0.03)	0.136
EPDS	1.30 (0.66–1.94)	< 0.001	0.42 (− 0.49–1.32)	0.365	2.29 (1.39–3.19)	< 0.001
Life events	1.80 (1.00–2.60)	< 0.001	2.97 (1.93–4.01)	< 0.001	− 0.02 (− 1.28–1.23)	0.971
Divorce	− 0.46 (− 1.15–0.22)	0.186	− 0.26 (− 1.21–0.69)	0.588	− 0.75 (− 1.74–0.23)	0.132
Unemployment	1.29 (0.61–1.98)	< 0.001	1.00 (0.10–1.91)	0.030	1.78 (0.73–2.84)	0.001
Parental immigration	0.42 (− 0.32–1.15)	0.265	0.40 (− 0.53–1.32)	0.397	0.53 (− 0.70–1.77)	0.394
Persistence (PS)	0.09 (− 0.05–0.22)	0.210	0.06 (− 0.13–0.26)	0.507	0.12 (− 0.08–0.31)	0.240
EPDS	1.23 (0.59–1.87)	< 0.001	0.34 (− 0.56–1.25)	0.457	2.23 (1.33–3.13)	< 0.001
Life events	1.75 (0.95–2.55)	< 0.001	2.93 (1.88–3.98)	< 0.001	− 0.07 (− 1.33–1.18)	0.911
Divorce	− 0.53 (− 1.22–0.15)	0.127	− 0.33 (− 1.28–0.63)	0.502	− 0.81 (− 1.79–0.17)	0.103
Unemployment	1.27 (0.58–1.95)	< 0.001	0.97 (0.07–1.87)	0.036	1.78 (0.73–2.83)	0.001
Parental immigration	0.41 (− 0.32–1.15)	0.270	0.38 (− 0.54–1.31)	0.418	0.59 (− 0.64–1.82)	0.348

*CI* confidence interval, *EPDS*  Edinburgh Postnatal Depression Scale. Multivariate linear regression. Dependent variables: CBCL internalizing scores. Independent variables: Maternal temperamental traits, EPDS, life events, divorce, unemployment, parental immigration status

**Table 5:** Maternal character traits predicting child internalizing problems in the total population and boys and girls respectively. Linear regression

	Total population		Boys		Girls	
	Adjusted B (95.0 % CI)	p-value	Adjusted B (95.0 % CI)	p-value	Adjusted B (95.0 % CI)	p-value
*Maternal character trait*						
Self-directedness (SD)	− 0.17 (− 0.23– − 0.12)	< 0.001	− 0.19 (− 0.27– − 0.11)	< 0.001	− 0.17 (− 0.25– − 0.09)	< 0.001
EPDS	0.68 (0.02–1.34)	0.045	− 0.36 (− 1.30–0.59)	0.456	1.79 (0.87–2.71)	< 0.001
Life events	1.72 (0.93–2.51)	< 0.001	2.92 (1.89–3.95)	< 0.001	− 0.14 (− 1.38–1.10)	0.827
Divorce	− 0.29 (− 0.97–0.39)	0.397	− 0.04 (− 0.99–0.90)	0.930	− 0.63 (− 1.60–0.34)	0.203
Unemployment	1.27 (0.59–1.94)	< 0.001	0.97 (0.08–1.86)	0.034	1.76 (0.72–2.80)	0.001
Parental immigration	0.29 (− 0.44–1.01)	0.439	0.31 (− 0.60–1.22)	0.503	0.34 (− 0.89–1.56)	0.590
Cooperativeness (CO)	− 0.18 (− 0.25– − 0.11)	< 0.001	− 0.16 (− 0.26– − 0.06)	0.001	− 0.20 (− 0.30– − 0.11)	< 0.001
EPDS	1.12 (0.48–1.75)	0.001	0.26 (− 0.64–1.16)	0.576	2.09 (1.19–2.98)	< 0.001
Life events	1.83 (1.03–2.62)	< 0.001	2.98 (1.95–4.02)	< 0.001	0.01 (− 1.22–1.25)	0.983
Divorce	− 0.30 (− 0.98–0.38)	0.386	− 0.12 (− 1.07–0.83)	0.801	− 0.56 (− 1.53–0.42)	0.263
Unemployment	1.34 (0.66–2.02)	< 0.001	1.00 (0.10–1.90)	0.029	1.91 (0.87–2.96)	< 0.001
Parental immigration	0.41 (− 0.31–1.14)	0.264	0.40 (− 0.52–1.32)	0.392	0.51 (− 0.71–1.73)	0.410
Self− transcendence (ST)	− 0.01 (− 0.07–0.06)	0.820	0.08 (− 0.08–0.10)	0.859	− 0.01 (− 0.10–0.08)	0.791
EPDS	1.27 (0.63–1.92)	< 0.001	0.37 (− 0.54–1.27)	0.430	2.28 (1.37–3.18)	< 0.001
Life events	1.79 (0.99–2.60)	< 0.001	2.95 (1.90–4.00)	< 0.001	− 0.02 (− 1.28–1.25)	0.977
Divorce	− 0.51 (− 1.20–0.17)	0.140	− 0.29 (− 1.25–0.66)	0.545	− 0.82 (− 1.80–0.16)	0.101
Unemployment	1.28 (0.60–1.97)	< 0.001	0.97 (0.07–1.88)	0.035	1.79 (0.74–2.84)	0.001
Parental immigration	0.43 (− 0,31–1.17)	0.250	0.39 (− 0.55–1.33)	0.414	0.57 (− 0.66–1.81)	0.362

*CI* confidence interval, *EPDS*  Edinburgh Postnatal Depression Scale. Multivariate linear regression. Dependent variables: CBCL internalizing scores. Independent variables: Maternal character traits, EPDS, life events, divorce, unemployment, parental immigration status

#### Gender specific analyses

Maternal novelty seeking was not associated with internalizing problems in boys or girls at age 3. Maternal harm avoidance was associated with internalizing problems in both boys and girls (Table 4). Maternal reward dependence and persistence were not associated with internalizing problems at age 3 in either boys or girls. Maternal character traits of self-directedness and cooperativeness were negatively associated with internalizing problems in both boys and girls while no association was found for self-transcendence (Table 5).

### Drop‐out rate analysis

The total dropout rate was 15.7 % (n = 271). There was a difference with respect to parental immigrant status between participants and non-participants, where 86.4 % (n = 1320) of mothers born in Sweden (n = 1527) took part compared to 71.6 % (n = 73) born in Europe (n = 102) and 62.8 % (n = 59) born outside Europe (n = 94) (χ2 = 50.654, p < 0.001). No significant differences were found between participants and non-participants at the 3 year-follow-up regarding symptoms of postpartum depression.

## Discussion

The aim of the present study was to investigate the associations of maternal temperament and character with externalizing and internalizing problems among children 3 years of age. The results could be summarized in four main findings:

Firstly, the temperamental trait of novelty seeking in mothers was associated with externalizing problems in 3-year-old children. This finding stands in contrast to the studies of Lee et al. (2015) and Yurumez et al. (2014) [8, 9]. However, novelty seeking have previously been shown to be associated with intraindividual externalizing type of problems such as alcohol inebriation in adolescents [27] and ADHD in the adult population [5]. The association between novelty seeking in mothers and externalizing problems in children could possibly represent child temperamental traits or behavioral patterns based on constitutional factors. Previous studies have shown an association between gene variants of DRD4 and novelty seeking, indicating associations to dopamine levels [28], and the heritability of novelty seeking has been shown to be comparably high [6]. However, environmental factors, including parenting behavior, are likewise possible mediators of the association found. Assessments of externalizing psychopathology in mothers would have been interesting to further analyze these associations. Interestingly, novelty seeking was associated with externalizing problems in girls, but not in boys. These gender differences should be further explored in future studies.

Secondly, maternal temperamental trait of harm avoidance was associated with both internalizing and externalizing problems in 3-year-old children. Harm avoidance has previously been associated with ADHD and behavior problems in young children [8, 9]. Whether this result reflects maladaptive child behaviors influenced by maternal harm avoidance, or whether mothers with the temperamental trait of harm avoidance are more prone to report behavioral problems in their children cannot be concluded using the present data-set. A high score on harm avoidance has previously been reported in adults with anxiety and other mental disorders [29, 30, 7], and a moderate correlation (r = 0.33) was found between harm avoidance and EPDS in the present data-set. Environmental influences and parental modeling shape the development of child behavior. For example, in line with the social learning theory of Bandura, children learn and adopt anxious behaviors from their parents. Parental modeling happens as the child observe anxious or avoidant behaviors, or when parents communicate their anxiety to others or directly to the child [31]. Parental reinforcement of child avoidant behaviors may also play a role in the development and maintenance of child anxiety [32]. Constitutional factors might also be at play, in a large twin study, high heritability was found for harm avoidance [33].

Thirdly, maternal character traits of self directedness and cooperativeness were negatively associated with both externalizing and internalizing problems in children. The same pattern was seen when analyses were run separately for boys and girls. In general, effect sizes on child behavior were greater for character traits than for temperamental traits. This finding is in line with Lee et al. (2015) [9], stating that parental character traits seem to impact child behavior to a greater extent than parental emotional style. Character traits are argued to reflect personal maturity and impact wellbeing and psychological functioning to a great extent [34], thus, potentially in turn impacting parenting style. Character traits mature over time and parenthood usually takes place during early adult life. Thus, interventions directed at parental support could be beneficial in families with significant difficulties. Parenting interventions could enhance consciousness about both own and child emotional and behavioral reactions, as well as promote the parent-child relationship.

Fourthly, while not being the main focus of the current paper, interesting gender differences with regard to vulnerability to included risk factors were found. Experience of life events was strongly associated with externalizing and internalizing problems in boys but not in girls. In fact, experience of life events had the greatest impact on externalizing and internalizing problems in boys of all included variables. Gender differences were also noted for maternal symptoms of postpartum depression, increasing the risk for externalizing and internalizing problems in girls but not in boys. This result stands in contrast to previous literature showing an increased risk for behavioral and developmental problems of boys whose mothers suffered from postpartum depression [35] and mental health problems [36]. However, adolescent girls have previously been shown to be more susceptible to depression if their mothers were depressed prenatally compared to boys [37]. Prenatal depression was, however, not assessed in the current study. Moreover, previous studies have indicated that sensitivity to adversity between boys and girls might vary with age [38, 39], with boys being more susceptible to stressful events in early childhood. Given gender differences in prevalence of psychiatric conditions, increased knowledge on the relative importance of risk factors during different developmental periods for boys and girls respectively, is of interest to further understand and outmost to prevent the development of mental health problems in children and adolescents.

Lastly, the associations between maternal temperamental and character traits and child behavior could be linked in different, non-exclusive ways. Firstly, genetic factors impact both temperament and behavior [40, 41]. Secondly, parental behaviors do not exert an unidirectional impact on child behavior, instead child temperament and response influence the child-parent interaction [14, 15]. Rettew et al. (2006) showed that the interaction between parent and child temperament dimensions predicted higher levels of child internalizing and externalizing problems than the traits alone [15]. Thirdly, as mentioned above, parental modelling shapes the development of child behavior, and multiple pathways and interaction patterns are most likely at play simultaneously.

### Limitations

The strengths of the present study are the large data set, the use of validated questionnaires and the longitudinal approach. However, the following limitations need to be taken into consideration. Firstly, child behavior at age 3 was reported only by the mothers, thus maternal mental health and temperamental profile could influence the way child behavior was observed, interpreted and reported. The extent to which maternal mental health effect reports on child behavior has been discussed, and findings indicate that informant discrepancies can be the result of both the context for symptom expression and individual informant factors [42]. Studies on 10–12-year-old children indicate that maternal depressive symptoms do not influence the results to a serious degree [43]. Moreover, at preschool age, child wellbeing can be argued to be best known by the primary caregiver. Secondly, there was no assessment of fathers’ mental health and temperamental profiles. The impact of parental temperament, character and mental health on child behavior and development depends on family constitution, and children are under the influence of all adults in the household. Assessment of fathers’ temperament would also have been interesting in light of genetic transmission. Thirdly, the retention rate at the 3-year follow-up was 84.3 %, and there was a significant difference in participation rate regarding parental country of origin. Loss to follow-up at similar rates are common in longitudinal studies [44], and previous studies indicate no notable differences in behavior problems for children of immigrants [45].

## Conclusions

Maternal character traits were more influential on child behavior problems than were temperamental traits, and thus the opportunities for intervention targeted at parental support are good. Other factors increasing the risk for child behavior problems are maternal mental health and socioeconomic aspects such as parental unemployment, indicating the need for recognition and support in clinical settings.

## Data Availability

Ethical Review Board approval was obtained for public sharing and presentation of data on group level only. This means that the data used in this study can only be used for the approved research and cannot be shared by the authors.

## Abbreviations

ADHD: Attention deficit hyperactivity disorder
CBCL: Child Behavior Checklist
CI: Confidence interval
CLES: Coddington Life Event Scale
CWC: Child Welfare Center
DRD4: Dopamine Receptor D4
EPDS: Edinburgh Postnatal Depression Scale
TCI: Temperament and Character Inventory
